# Exciton Transport in the Nonfullerene Acceptor O-IDTBR from Nonadiabatic Molecular Dynamics

**DOI:** 10.1021/acs.jctc.4c00605

**Published:** 2024-07-05

**Authors:** Ljiljana Stojanovic, Samuele Giannini, Jochen Blumberger

**Affiliations:** † Department of Physics and Astronomy and Thomas Young Centre, University College London, London WC1E 6BT, U.K.; ‡ Institute of Chemistry of OrganoMetallic Compounds, National Research Council (ICCOM-CNR), Pisa I-56124, Italy

## Abstract

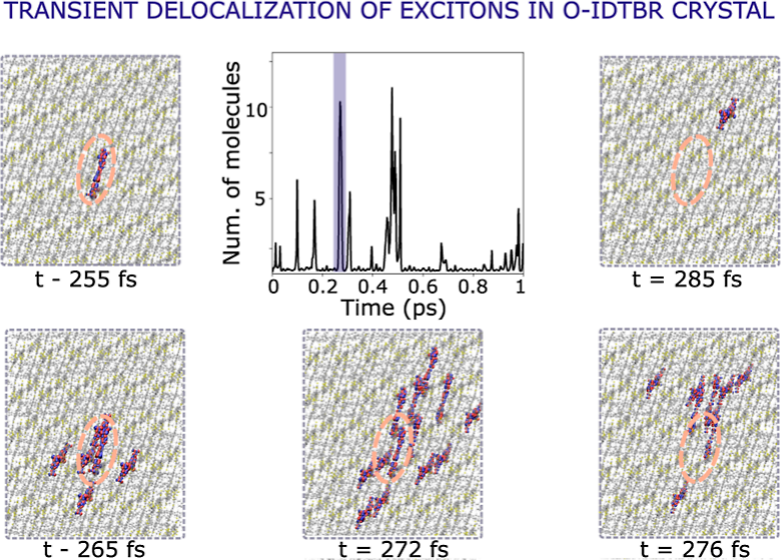

Theory, computation, and experiment have given strong evidence that charge carriers in organic molecular crystals form partially delocalized quantum objects that diffuse very efficiently via a mechanism termed transient delocalization. It is currently unclear how prevalent this mechanism is for exciton transport. Here we carry out simulation of singlet Frenkel excitons (FE) in a molecular organic semiconductor that belongs to the class of nonfullerene acceptors, O-IDTBR, using the recently introduced FE surface hopping nonadiabatic molecular dynamics method. We find that FE are, on average, localized on a single molecule in the crystal due to sizable reorganization energy and moderate excitonic couplings. Yet, our simulations suggest that the diffusion mechanism is more complex than simple local hopping; in addition to hopping, we observe frequent transient delocalization events where the exciton wave function expands over 10 or more molecules for a short period of time in response to thermal excitations within the excitonic band, followed by de-excitation and contraction onto a single molecule. The transient delocalization events lead to an increase in the diffusion constant by a factor of 3–4, depending on the crystallographic direction as compared to the situation where only local hopping events are considered. Intriguingly, O-IDTBR appears to be a moderately anisotropic 3D “conductor” for excitons but a highly anisotropic 2D conductor for electrons. Taken together with previous simulation results, two trends seem to emerge for molecular organic crystals: excitons tend to be more localized and slower than charge carriers due to higher internal reorganization energy, while exciton transport tends to be more isotropic than charge transport due to the weaker distance dependence of excitonic versus electronic coupling.

## 1 Introduction

The transport of bound electron–hole pairs or excitons^1^ is essential to the function of organic optoelectronic materials in light-emitting diodes and solar cells. The simplest theoretical models describing excited state transport in organic semiconductors (OS) are rate theories that have been originally developed for molecular donor–acceptor systems, for example, Marcus–Levich–Jortner.^2^ Structurally disordered organic materials behave in certain respects similarly to molecular–donor–acceptor systems and therefore the application of rate theories to these materials has been successful.^3^ However, the application of these theories to situations that fall outside their well-defined regimes of validity is problematic. For instance, donor–acceptor rate theories are bound to fail qualitatively for highly ordered or single-crystalline OS. This is because they do not take into account the excitonic band structure of the extended system and instead consider only the excitonic interaction between a single pair of molecules at a time. Moreover, the transport rates in these materials may be faster than typical molecular relaxation times violating the assumption of (quasi-) thermal equilibrium inherent to most rate theories. Thus, direct quantum dynamical simulations of the coupled electron–nuclear motions that account for such phenomena are highly desirable, for instance multiconfigurational time dependent Hartree method^4−6^ or computationally more tractable trajectory-based nonadiabatic dynamics methods.^7−11^

Recent computational and experimental studies of charge transport in ordered OS have shown strong evidence that holes or excess electrons form partially delocalized quantum objects “half way” between waves and particles.^12−29^ The partial delocalization is a consequence of the specific interplay among important transport parameters such as reorganization energy, electronic couplings and their thermal fluctuations. Specifically, when the reorganization energy is much larger than twice the electronic coupling values, the charge is fully localized on a single site and it has to overcome a potential barrier in order to transfer from one site to another.^19^ In such a case, thermal fluctuations in electronic couplings will enhance mobility through a phonon-assisted transport mechanism as predicted by Munn and Silbey^25^ and further demonstrated by explicit nonadiabatic dynamics simulations.^16,30^ On the other hand, when reorganization is smaller than twice the electronic coupling, the charge carrier tends to delocalize over multiple sites depending on the strength of the interactions. This is often the case in application-relevant high-mobility OS.^17,19^ In this regime, however, the ubiquitous and sizable intermolecular vibrations between the weakly bound molecules in OS give rise to large thermal fluctuations of electronic coupling that prevent full wave-like delocalization.^17,19^ The partially delocalized charge carriers were found to diffuse through OS via a transient delocalization mechanism^17−21,25,28,29^ in a scenario that cannot be described with the standard tools developed for hopping transport in donor–acceptor systems or band transport in inorganic materials.

While numerous examples for charge transport in the transient delocalization regime have been reported,^12−14,17−21,25,28,29^ it is less clear to which extent this mechanism carries over to exciton transport in highly ordered OS.^6,10,31^ As discussed and explained in ref (10 and 32), singlet excitons typically have larger internal (or “inner sphere”) reorganization energies than charge carriers because the change of bonding to antibonding interactions and vice versa when the electron is excited from occupied to unoccupied orbitals gives rise to large changes in the bond lengths.^10,32^ This effect favors exciton localization. On the other hand, the excitonic couplings between singlet exciton states are long-range dipolar interactions, in contrast to electronic couplings which decay exponentially with distance as they are proportional to orbital overlap on neighboring molecules.^10,31^ In addition, thermal fluctuations of excitonic couplings tend to be much smaller than for electronic couplings because they are no longer exponentially sensitive to distance.^10,31^ The last two trends would favor exciton delocalization. We note in passing that, in contrast to singlets, triplet exciton transitions are spin-forbidden and the corresponding excitonic couplings are short-range because the dipolar interaction term that is dominating for singlets goes to zero. Consequently, for triplet excitons one would expect transport behavior more alike that of an excess charge.

It is now important to study exciton transport in a diverse range of OSs to obtain a better understanding of the underlying physical mechanism that will be beneficial for the design of improved optoelectronic materials. Here we characterize singlet exciton transport in O-IDTBR, an OS that belongs to the class of nonfullerene acceptor materials (NFA) that have boosted power conversion efficiency of organic solar cells to >19%.^33,34^ O-IDTBR has a large extinction coefficients and a wide absorption range leading to efficient exciton generation.^35^ It exhibits good photostability in organic solar cells with a range of donor materials and is less prone to photodegradation^36,37^ and recombination losses^38,39^ compared to some other families of NFAs. In combination with donor polymers in bulk heterojunctions, O-IDTBR binary blends enable power conversion efficiencies above 10%^40^ and significant efficiencies with small molecule donors.^39^ In ternary cells with the NFA Y6, power convergence efficiencies of 16.6% were reported.^41^ Apart from the applications in photovoltaics, O-IDTBR is also suitable as an n-type material in organic thin-film transistors.^42^

We investigate O-IDTBR because it is very well suited for fundamental studies of exciton diffusion. First, this material exhibits long exciton lifetimes, ≈0.6 ns,^38,43^ indicating small quantum efficiencies of competing deactivation channels, such as fluorescence, intersystem crossing,^44^ or generation of intermolecular charge-transfer states. Second, the X-ray crystallographic structure of O-IDTBR single crystals is available.^42^ Third, charge transport in O-IDTBR has been previously studied in our group using nonadiabatic molecular dynamics simulation.^45^ Hence, the current study enables us to understand similarities and differences between exciton and charge carrier transport in the same material on an equal footing. We find evidence that although the exciton is on average fully localized on a single molecule in O-IDTBR single crystals, the diffusion mechanism is not simply local hopping but strongly enhanced by frequent transient delocalization events. Moreover we find that O-IDTBR single crystals are 3D “conductors” for excitons but 2D conductors for charge carriers.

In the following section, our previously introduced Frenkel exciton surface hopping (FE-SH) nonadiabatic dynamics method is reviewed and details on the parameterization of the Frenkel exciton Hamiltonian from TDDFT calculations and the simulation protocol are given. In the Results and Discussion section, the S_1_ excited state and the excitonic couplings in the crystal are characterized before the main results of this paper are presented, the dynamics and mechanism of exciton diffusion in the O-IDTBR crystal, as obtained from FE-SH. The results obtained are then discussed in section Discussion in light of previous simulations on related OSs and experimental measurements. Moreover, parallels and differences between singlet exciton and electron transport are highlighted. Our work is summarized in the Conclusions section.

## 2 Methods

### 2.1 Frenkel Exciton Surface Hopping

Exciton transport in O-IDTBR crystals is simulated using FE-SH.^10^ In this method, the Frenkel exciton (FE) Hamiltonian is constructed in the space of quasi-diabatic or localized excitonic states,  = 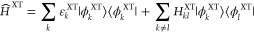
1where **r** describes the position of the exciton,  is the site energy of site (or molecule) *k*, that is, the total potential energy of the system with site *k* in the excited state and all other sites in their electronic ground state, and  are the excitonic couplings between two localized excitonic states,  and . The localized excitonic states as well as the site energies and excitonic couplings depend on the coordinates of the (classical) nuclei, **R**, which depend on time *t*, **R** = **R**(*t*). In this way, diagonal and off-diagonal exciton–phonon coupling are incorporated.

The exciton wave function, Ψ(*t*), is expanded in the basis spanned by the localized excitonic states used to represent the FE Hamiltonian
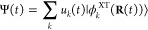
2where *u*_*k*_(*t*) are the expansion coefficients. This form of the wave function is inserted into the time-dependent Schrödinger equation to obtain a system of coupled time-dependent equations, describing the dynamics of the exciton wave function
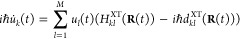
3where  are nonadiabatic coupling elements between localized excitonic states *k* and *l*. This equation has the same form as the equation describing the excess charge dynamics in the FOB-SH method.^15,46^ The second term on the right-hand side of eq 3 containing the nonadiabatic coupling between the quasi-diabatic states is typically small and can be neglected (see also ref (10)).

The classical nuclei are propagated on a single adiabatic excitonic state (referred to as “active state”), which is obtained by diagonalization of the FE Hamiltonian eq 1. Nonadiabatic transitions from the potential energy surface of the active adiabatic excitonic state to other adiabatic excitonic states are determined according to Tully’s surface hopping probability.^47^ In this way, feedback from the excitonic to the nuclear dynamics is incorporated. As in previous work, the original surface hopping methodology needs to be supplemented with a decoherence correction which is detailed further below in the Simulation Details section.

### 2.2 Exciton Diffiusion Constant

The mean-square displacement of the excitonic wave function, MSD_αβ_, is calculated as follows^10^
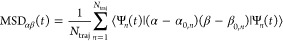
4
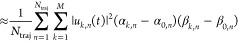
5where α and β are the Cartesian coordinates of the excitonic wave function Ψ_*n*_(*t*) and α_0,*n*_ the initial position in trajectory *n*, α_0,*n*_ = ⟨Ψ_*n*_(0)|α|Ψ_*n*_(0)⟩. The product of displacements is averaged over the total number of FE-SH trajectories, *N*_traj_. In eq 5, *u*_*k*,*n*_ are the expansion coefficients of Ψ_*n*_(*t*) in the localized excitonic basis (eq 2) in trajectory *n* and the coordinates of the exciton are replaced by the center of mass of molecule *k* in trajectory *n*, α_*k*,*n*_.

As presented in the Results section, after the initial relaxation period, Ψ(*t*) enters a diffusive regime, where the MSD components increase approximately linearly with time. In this regime, linear fits of the MSD components give the diffusion tensor via the Einstein relation
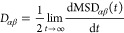
6

Exciton diffusion lengths (*L*_αβ_) can be computed based on the diffusion tensor elements (*D*_αβ_) as , where τ is the exciton lifetime, usually obtained from photoluminescence experiments.^48^

The extent of spatial delocalization of the exciton wave function is characterized by the inverse participation ratio (IPR), which is a measure for the number of molecules over which the wave function is delocalized^10^
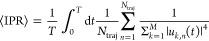
7where *M* is the number of molecules included in the FE-SH dynamics and *T* is the length of the trajectories.

### 2.3 Parameterization of the FE Hamiltonian

#### 2.3.1 Excitation and Reorganization Energy

We computed the first three singlet excitation energies (S_1_, S_2_, S_3_) and the S_1_ reorganization energies of a single O-IDTBR molecule in a vacuum with three different hybrid density functionals, CAM-B3LYP,^49^ ωB97X-D,^50^ and M06-2X,^51^ applying the TDDFT method. The range-separated hybrid functionals CAM-B3LYP and ωB97X-D are commonly used for the description of localized and charge-transfer excited states in organic molecules,^10^ whereas the global hybrid functional M06-2X functional has been previously used for the calculation of excited states on a set of nonfullerene acceptors.^52^ All calculations were carried out with the Gaussian program^53^ using the 6-31G(d,p) basis set. We obtained similar results for these functionals, see the Supporting Information for a summary. We chose CAM-B3LYP for parameterization of the FE Hamiltonian to remain as consistent as possible with the parameterization of the force field for ground state O-IDTBR carried out in previous work^54^ (also see below).

#### 2.3.2 Site Energies

The diagonal element of the FE Hamiltonian, also denoted as site energy,  in eq 1, is the electronic energy of the system with the molecule *k* in the S_1_ excited state and all other molecules in their S_0_ electronic ground state. The description of the molecules in the electronic ground state is based on a classical force field previously parameterized for O-IDTBR using B3LYP/6-311G(d,p) calculations.^54^ Since we apply the CAM-B3LYP functional in this work, some of the intramolecular interactions of the original O-IDTBR force field were modified and made consistent with the CAM-B3LYP functional. In particular, we found that the equilibrium distances of the bonding interactions at CAM-B3LYP/6-31G(d,p) level differ somewhat from the ones at B3LYP/6-311G(d,p) level, hence they were modified to match the CAM-B3LYP/6-31G(d,p) values. By contrast, the differences in equilibrium values for bending and dihedral interactions were negligibly small and remained unchanged. For parameterization of the molecules in the S_1_ excited state, the equilibrium bond lengths were adjusted so that the intramolecular reorganization energy λ_intra_ of O-IDTBR in vacuum obtained from the force field matches the corresponding value obtained from the TDDFT calculations at CAM-B3LYP/6-31G(d,p) level
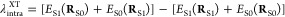
8where *E*_S0_ and *E*_S1_ are the potential energy of the molecule in the S_0_ ground and S_1_ excited state, respectively, and **R**_S0_ and **R**_S1_ are the nuclear coordinates at the minimum of the S_0_ and S_1_ potential energy surfaces. We obtained a value  = 330 meV.

The site energies include only intramolecular terms as explained above and Lennard–Jones interactions (taken to be the same in ground and excited state) but they do not contain electrostatic interactions as they would be prohibitively expensive to compute for all the *M* site energies during FE-SH propagation. In previous work we have shown using classical molecular dynamics simulation with the original force field for O-IDTBR^54^ that the average electrostatic potential on each of the 4 molecules in the unit cell is the same within statistical error bars. Hence, there is no static electrostatic disorder among the different sites in the crystal. The dynamic electrostatic disorder in the O-IDTBR crystal was previously estimated to correspond to an intermolecular or external reorganization energy for electron transfer of 80 meV. For exciton transfer, this intermolecular contribution, , is expected to be even smaller since the crystal environment reorganizes in response to a change in transition dipole, not to a shift in charge. Thus, the total reorganization energy is λ^XT^ ≈ . These considerations imply that neglecting the intermolecular electrostatic interactions during FE-SH and the associated intermolecular reorganization energy should be a reasonably good approximation.

#### 2.3.3 Excitonic Couplings

The off-diagonal matrix elements of the FE Hamiltonian, also denoted as excitonic couplings  in eq 1, are approximated by the excited state couplings of molecular pairs in vacuum, *V*_*kl*_. The excitonic coupling between singlet exciton states includes terms originating from the long-range electrostatic contributions and the short-range interactions (exchange–correlation, orbital overlaps, and electronic polarization effects). We have previously shown on a set of pi-conjugated organic dimers, that the short-range interactions have a negligible contribution to the couplings even in the case of molecular crystals where the molecules are in close contact, and that the excitonic couplings are very well approximated by the Coulomb contribution only^10^
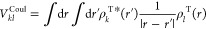
9where  and  are the transition densities for the S_1_ excited state on the molecules *k* and *l*, respectively.

During FE-SH dynamics several thousand excitonic couplings need to be computed at run time to construct the FE Hamiltonian eq 1 at each MD time step. Hence, a fast method is required to evaluate the Coulomb integral eq 9. Here it is approximated by representing the transition densities in terms of atom-centered transition charges, using the transition electrostatic potential charges (TrESP) prescription^55^
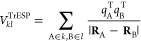
10where the indices A and B run over the atoms for molecules *k* and *l*, respectively,  and  are the TrESP charges and **R**_A_ and **R**_B_ are the positions of atoms A and B, respectively. The TrESP charges are calculated for the minimum energy structure of a single O-IDTBR molecule and frozen during FE-SH dynamics, thereby avoiding the need to perform electronic structure calculations for each pair at each time step. The TrESP method not only provides accurate electronic couplings through a straightforward Coulomb sum (see the Results and Discussion section), but crucially, it also maintains the consistency of the coupling signs across all different molecular pairs during FE-SH dynamics.

### 2.4 Simulation Details

We performed FE-SH simulations on three crystal planes—*ab*, *ac*, and *bc*—using supercells of dimensions 8 × 1 × 8, 1 × 8 × 8 and 8 × 8 × 1. The supercells are built from the experimental crystal structure of O-IDTBR.^42^ For each supercell an ensemble of excited states is prepared where a single molecule *i* in the center of the supercell is in the S_1_ excited state and all other molecules in the ground state as described by the modified force field for O-IDTBR (see above for details of parameterization). The atomic positions are first relaxed to their local minimum energy configuration and subsequently equilibrated to 300 K by running classical MD simulations of the excited state for 0.5 ns in the *NVT* ensemble applying periodic boundary conditions. The final configurations and velocities from these runs were used to initialize MD trajectories in the *NVE* ensemble, run for 0.5 ns. The structure of the O-IDTBR crystal was stable during these runs, rmsd = 0.7 Å with respect to the crystal structure. From the last 100 ps of the *NVE* run 100 equidistantly spaced structures and velocities were taken and used as initial coordinates for FE-SH dynamics in the *NVE* ensemble. The FE-SH simulations were performed for rectangular active regions in the *ab*, *ac*, and *bc* crystallographic planes of dimensions 6 × 6 containing 144 molecules, which we refer to as “electronically active” molecules because they are used for the construction of the FE-SH Hamiltonian. The remaining molecules (composed of 1 molecular layer embedding the active region) are “electronically inactive” but interact with the electronically active molecules via their nonbonded force field terms to ensure the structural integrity of the latter. The exciton dynamics in the electronically active region is not periodically repeated, i.e., there are no interactions of the excitons with periodic images.

In accordance with the prepared initial configurations, the initial exciton wave function was chosen to be localized on the central molecule *i*, Ψ(0) = . The wave function and the nuclei were propagated in time running FE-SH in the *NVE* ensemble, applying decoherence correction, specifically, exponential damping of the amplitudes of nonactive excitonic band states with a Heisenberg decoherence time, removal of the spurious long-range exciton transfer transitions, trivial crossing detection and adjustment of the velocities in the direction of the nonadiabatic coupling vector after a successful hop to preserve total energy, see refs (16, 46, 56, and 57) for details. As shown in refs (16, 46, 56, and 57) these algorithms are necessary to avoid unphysical long-range transfer events and to guarantee convergence of the diffusion coefficient in large systems with a high-density of states. The nuclear time step was 0.02 fs in all simulations and a multiple time step algorithm for the update of the excitonic Hamiltonian matrix elements was used as detailed in ref (18). Integration of eq 3 was carried out using a fourth-order Runge–Kutta algorithm with an electronic time step that was 1/5 of the nuclear time step in each case. The number of simulated trajectories was *N*_trj_ = 300 for each supercell, each of length 1 ps. All MD and FE-SH simulations were done with an in-house implementation of FE-SH in the CP2K program package.^58^

## 3 Results and Discussion

### 3.1 Crystal Structure and Excited States

**Figure 1:**
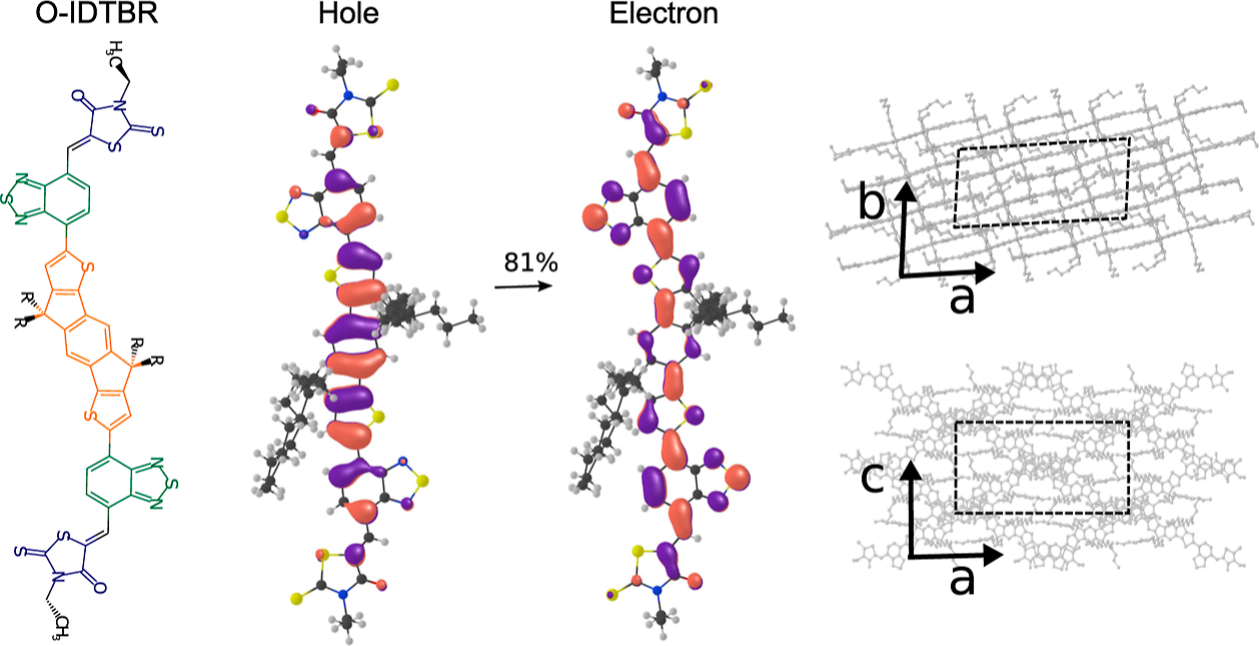
Chemical structure of O-IDTBR (left panel). The RH group is shown in blue, the BT group in green, and the IDT group in orange. *R* represents the octyl group. Natural transition orbitals (NTOs) giving the largest contribution (81%) to the S_1_ singlet excited state (middle two panels). Two projections along the *a*–*b* and *a*–*c* planes of the experimental crystal structure of O-IDTBR are shown in the right panel. The unit cell is indicated in dashed lines. The crystal structure was taken from ref (42) defining the crystallographic axes as shown, in line with our previous work in ref (45).

The chemical structure and the crystal structure of O-IDTBR are shown in Figure 1. O-IDTBR is an A–D–A type nonfullerene acceptor, with the central indacenodithiophene (IDT) group an electron-rich donor connected to the electron-accepting benzothiadiazole (BT) and rhodanine (RH) groups. Four molecules arrange in a monoclinic unit cell in an interdigitated columnar structure.^35,42^

NFAs with the A–D–A structure are usually characterized by a bright S_1_ state, responsible for the efficient photon absorption in the region between 600 and 700 nm in photovoltaic devices.^59,60^ The NTOs, obtained by the diagonalization of the transition density matrix, provide a compact representation of the excited state in terms of a small number of single–particle transitions. At the TDDFT level used in this study [CAM-B3LYP/6-31G(d,p)], a pair of NTOs with the most significant contributions to the S_0_ → S_1_ transition (81%) are π-conjugated orbitals delocalized mainly over IDT and BT groups (see Figure 1). These NTOs resemble very closely the HOMO and LUMO (see the Supporting Information), the main difference being the smaller degree of delocalization of the NTOs over the RH groups. The S_1_ state is characterized by a large transition dipole moment aligned along the longest molecular axis, while the second singlet excited state (S_2_) is a dark state, ca. 0.35 eV above S_1_.

In the O-IDTBR crystal, the intramolecular S_1_ excited states linearly combine to form the S_1_ excitonic band and similarly for S_2_. The sizable energetic separation of intramolecular S_1_ and S_2_ states imply that the low energy tail of the S_1_ excitonic band is well separated from the S_2_ excitonic band. This implies that the low excitation energy dynamics simulated herein is well described by the low energy tail of the S_1_ excitonic band, in line with the common FE approximation^1^ and justifying the use of a FE Hamiltonian eq 1 in the current study. Low-lying intermolecular charge transfer states and triplet states may be present in O-IDTBR and are neglected in our current treatment but could be included in a future extension of our method by expanding the state space of the electronic Hamiltonian.^5^

### 3.2 Excitonic Couplings

**Figure 2:**
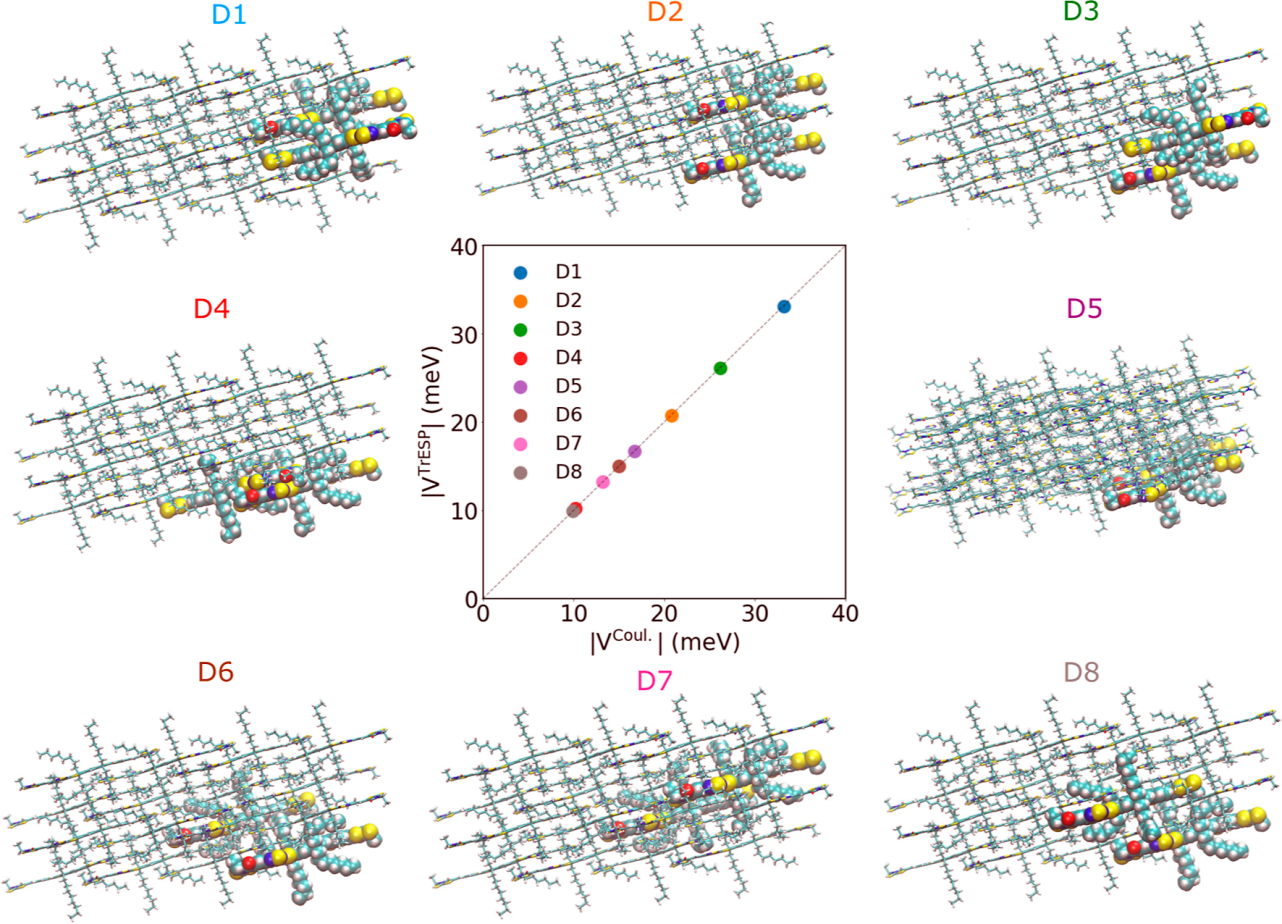
Excitonic couplings in the O-IDTBR crystal. Atoms of dimers with the strongest couplings, *D*_1_–*D*_8_, are enlarged and shown as spheres within a 2 × 2 × 2 supercell. A scatterplot  vs  for *D*_1_–*D*_8_ dimers in their crystallographic positions is shown in the middle. Note the excellent approximation of the Coulomb couplings *V*^Coul^eq 9 by the transition charge couplings *V*^TrESP^eq 10.

Excitonic couplings for molecular pairs in the crystal structure with a magnitude ≥10 meV are summarized in Table 1. The corresponding dimer structures are shown in Figure 2. We find that the TrESP couplings calculated for each dimer are nearly indistinguishable from the Coulomb couplings. The relative error is <0.5% in all cases attesting to the accuracy of the TrESP approach. The largest couplings are relatively modest, between 20 and 30 meV. All of the larger coupling values occur between molecules that are not immediate neighbors in the crystal, with the exception of dimer *D*_4_, in line with their long-range nature of excitonic couplings. Dimers *D*_2_, *D*_5_, *D*_6_, *D*_7_, and *D*_8_ have parallel main axes and aligned transition dipole vectors, whereas the rest of the dimers have in-plane rotated axes. All dimers except *D*_2_ and *D*_5_ contribute to exciton transport in the *a*-direction, all dimers except *D*_4_ and *D*_5_ contribute to transport in the *b*-direction and all dimers except *D*_4_ and *D*_2_ contribute to transport along the *c*-direction.

**Table 1:** Summary of Excitonic Couplings for Dimers in the O-IDTBR Crystal, All Values are in meV

dimer^a^	*d*_com_^b^ (Å)	c^,^^e^	*V*_*kl*_^Coul^^d^^,^^e^	f	f	σ(*V*_*kl*_^TrESP^)^g^	σ(*V*_*kl*_^Coul^)^g^	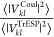
*D*_1_	10.1	–33.1	–33.2	27.6	28.2	0.9	3.8	1.06
*D*_2_	13.8	20.7	20.8	21.7	21.8	0.4	1.8	1.01
*D*_3_	11.2	–26.1	–26.2	28.3	29.1	0.7	3.1	1.06
*D*_4_	16.4	–10.2	–10.2	10.2	9.7	1.2	4.6	1.10
*D*_5_	15.8	16.7	16.7	18.0	17.4	0.4	3.0	1.09
*D*_6_	17.3	–15.0	–15.0	15.6	14.4	0.5	2.0	1.01
*D*_7_	18.0	–13.2	–13.2	15.3	15.1	0.3	3.1	1.01
*D*_8_	17.7	–9.9	–9.9	8.6	10.1	0.5	4.0	1.58

a See Figure 2.

b Center of mass distance between the monomers of a dimer.

c Excitonic couplings calculated in the transition charge (TrESP) approximation, eq 10, for dimers in the crystal structure configuration.

d Excitonic couplings calculated in the Coulomb approximation, eq 9, for dimers in the crystal structure configuration.

e The sign of the transition density and TrESP charges are defined consistently and in the same way on each molecule. This definition is arbitrary, thus a negative (positive) coupling sign does not mean *J*- (*H*-) aggregation in this context.

f Average TrESP or Coulomb excitonic couplings obtained along a MD trajectory of the crystal at 300 K.

g Root-mean-square fluctuations of TrESP or Coulomb excitonic couplings obtained along a MD trajectory of the crystal at 300 K.

To probe the effect of thermal fluctuations, we calculated excitonic couplings for *D*_1_–*D*_8_ dimers for 20 structures sampled from an equilibrated *NVE* trajectory of the crystal at 300 K at a spacing of 1 ps. The mean unsigned error (MUE) between TrESP and Coulomb couplings are <1 meV, and the mean relative unsigned error (MRUE) is less than 15% for all dimers, except for dimer *D*_4_ (MRUE = 35.3%) and *D*_8_ (MRUE = 39.9%). The most significant difference between TrESP and Coulomb couplings is in their thermal fluctuations. The TrESP couplings exhibit very small root-mean-square fluctuations, σ (mostly below 1 meV, see Table 1), whereas the fluctuations of the Coulomb couplings are substantially larger, typically a few meV. The main reason for this mismatch is the frozen TrESP charge approximation used for the calculation of the TrESP couplings. The TrESP charges are computed at the geometry extracted from the crystal, where the O-IDTBR molecules are relatively planar (dihedral angles between IDT and BT units less than 5°). The planarity of the molecule allows for delocalization of the S_1_ state transition density over IDT and BT groups. At room temperature, the strong dihedral motions can partially break pi-conjugation in O-IDTBR altering the S_1_ transition density distribution. This leads in some configurations to a significant reduction in delocalization of the transition density and an increased localization over the IDT units. This effect is not captured in the frozen TrESP charge approximation leading to the underestimation of the thermal fluctuations of excitonic couplings.

In the following, we estimate the error introduced by the underestimation of excitonic coupling fluctuations with frozen TrESP charges. We assume that exciton transport occurs in the nonadiabatic hopping regime, which is not strictly the case here as we will see further below, but a reasonable proxy to estimate the effect of underestimated coupling fluctuations on transport. Since according to Marcus theory, nonadiabatic hopping rates are proportional to the thermal average of the square of the couplings, ⟨|*V*_*kl*_|^2^⟩ = ⟨|*V*_*kl*_|⟩^2^ + σ^2^, the hopping rates increase with increasing coupling fluctuations. However, in case of O-IDTBR the coupling fluctuations are an order of magnitude smaller than the average couplings, even at the level of the full Coulomb couplings. Thus, they contribute only very little to ⟨|*V*_*kl*_|^2^⟩. Indeed, the ratio  is ≤1.1 for all dimers, except for the weakly coupled *D*_8_, where this ratio is 1.6. Thus, the error in the exciton transfer rates due to the inaccuracies in the frozen TrESP charge approximation is expected to be very small, ≤10% (see Table 1).

### 3.3 Exciton Diffusion

As detailed above, excitonic couplings are sizable along all three crystallographic directions. Hence, a large 3D sample of the crystal would need to be simulated to obtain the full exciton diffusion tensor, but unfortunately this is computationally prohibitive. Instead we chose to perform FE-SH simulations on three molecular planes—*ab*, *ac*, and *bc*—using supercells that have large dimensions along the in-plane directions (8 × 8) and only one unit cell along the out-of-plane direction. We verified that the delocalization of the exciton along the out-of-plane direction is sufficiently small so that one unit cell was sufficient for a faithful description of the exciton. Thus, each of the three simulations gives diffusion constants along the two in-plane directions but not along the out-of-plane direction because the supercell along this direction is too small to allow for the calculation of a diffusion constant.

**Figure 3:**
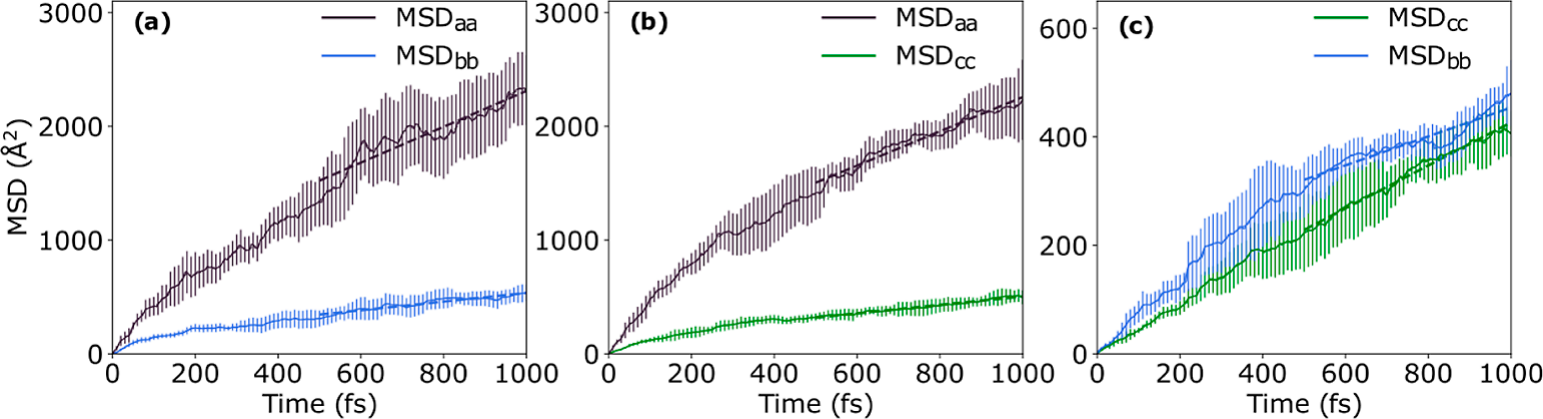
Exciton diffusion in O-IDTBR crystal. The MSDs eq 5 of the exciton wave function eq 2 are shown as a function of time, as obtained from FE-SH dynamics performed for the *a*–*b* (a), *a*–*c* (b), and *b*–*c* (c) crystallographic planes. Root-mean-square deviations of the MSD for the swarm of surface hopping trajectories are indicated by error bars. The MSDs are fit to straight lines between 0.5 and 1 ps to obtain the diffusion coefficients according to eq 6. Only the diagonal elements of the MSDs are shown.

The initial conditions for FE-SH dynamics were prepared such that the excitonic wave function was localized on one of the central molecules (index *i*) in the supercells, Ψ(0) = , see the Simulation Details section. The MSD components eq 5 are calculated along the crystallographic directions (α, β = *a*, *b*, *c*) and the time dependence of the two diagonal in-plane components are shown in Figure 3. In the first ≈200 fs of dynamics the initially localized excitonic wave function relaxes fairly rapidly and after about ≈500 fs of dynamics a stable diffusive regime is reached. We note that while the short-time dynamics depends on the initial conditions chosen, the dynamics in the diffusive regime is independent of initial conditions.^10^ The mean IPR averaged over trajectories and time (eq 7) is ⟨IPR⟩ = 1.2 indicating that most of the time the exciton remains localized on a single molecule, except when exciton transfer events occur as further analyzed in the Diffusion Mechanism section. The pronounced localization of the exciton is due to the relatively large reorganization energy λ^XT^ ≈  = 330 meV, which is about an order or magnitude larger than the largest excitonic couplings.

The MSD components are fit to straight lines in the diffusive regime at times >500 fs and the diffusion tensor *D*_αβ_ is calculated for each plane according to eq 6. The diagonal elements, *D*_*a*_ ≡ *D*_*aa*_, *D*_*b*_ ≡ *D*_*bb*_, *D*_*c*_ ≡ *D*_*cc*_, are summarized in Table 2. We note they are almost equal to the eigenvalues of the diffusion tensors since off-diagonal elements are small. Moreover, we notice that the FE-SH simulations for the three planes give two independent estimates for the diffusion coefficient along a particular direction that are in reasonably good agreement with one another. For further discussion we consider the average of the two values.

**Table 2:** Summary of Frenkel Exciton Diffusion Constants and Diffusion Lengths in O-IDTBR Crystal, in Units of 10^–3^ cm^2^ s^–1^ and nm, Respectively

plane	*D*_*a*_^a^	*D*_*b*_^a^	*D*_*c*_^a^	*D*′_*a*_^b^	*D*′_*b*_^b^	*D*′_*c*_^b^	*L*_*a*_^c^	*L*_*b*_^c^	*L*_*c*_^c^	*L*′_*a*_^d^	*L*′_*b*_^d^	*L*′_*c*_^d^
*a*–*b*	79	19		24	6.0		95	46		52	26	
*a*–*c*	87		7.2	19		3.0	99		28	47		18
*b*–*c*		13	20		6.4	6.9		39	47		27	28
average	83	16	14	22	6.2	5.0	97	42	38	49	26	23

a Diffusion constants along the crystallographic directions as obtained from eq 6, *D*_*a*_ ≡ *D*_*aa*_, *D*_*b*_ ≡ *D*_*bb*_, *D*_*c*_ ≡ *D*_*cc*_.

b Diffusion constants with transient delocalization events excluded, see text for details.

c Diffusion lengths , α = *a*, *b*, *c*, τ_exp_ = 561.5 ps.^43^

d Diffusion lengths with transient delocalization events excluded, see text for details.

We find that exciton diffusion is anisotropic, the largest diffusion constant is along the *a*-direction, *D*_*a*_ = 83 × 10^–3^ cm^2^ s^–1^, which is approximately five times larger than along the *b*- and *c*-directions. The corresponding exciton diffusion lengths, , α = *a*, *b*, *c*, for τ_exp_ = 561.5 ps,^43^ are 97 nm along the *a*-direction and about half that value along *b* and *c* (see Table 2). The anisotropy of the diffusion constants is somewhat surprising given that the excitonic couplings are fairly isotropic and the center of mass distances between the strongest coupled dimers *D*_1_–*D*_8_ are fairly similar, too. We explain this by the particular sign combination of the excitonic couplings (Table 1), which leads to formation of excitonic band states that are more delocalized along *a* than along *b* or *c*. The excitonic wave function Ψ(*t*) is composed of these excitonic bandstates and hence also tends to be more delocalized along *a* than along *b* or *c*, in particular during the transient delocalization events that contribute to diffusion (see below). Since the diffusivity is proportional to the exciton delocalization and the center of mass distance between the molecules,^10^ the diffusion constant along *a* is larger than in the other directions.

### 3.4 Diffusion Mechanism

**Figure 4:**
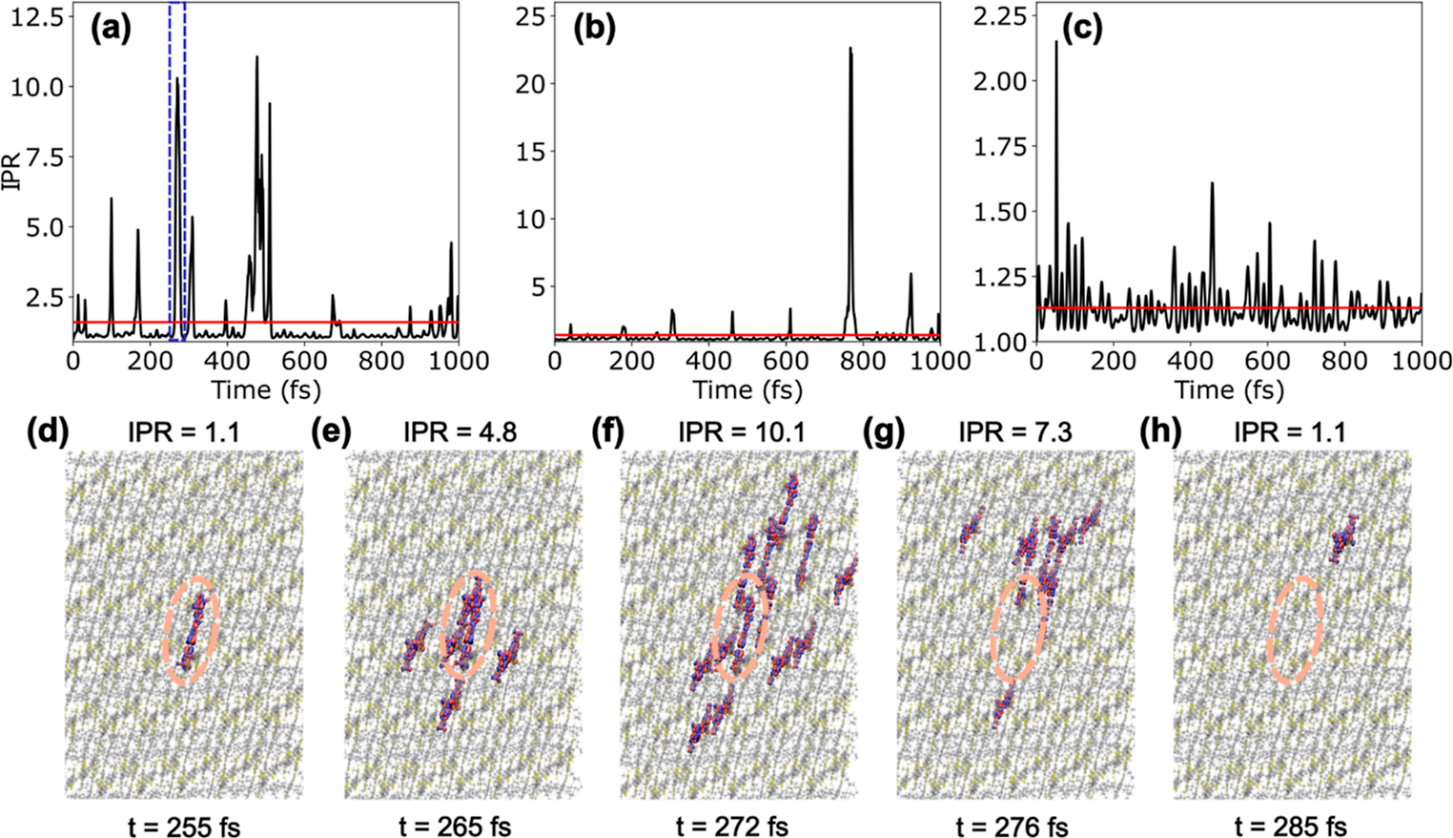
Transient delocalization of excitons in O-IDTBR. The IPR eq 7 as a function of simulation time is shown for three representative FE-SH trajectories (a–c), with mean IPR values indicated by red horizontal lines. A transient delocalization event along the trajectory in (a) between 255 and 285 fs (indicated by a rectangle in dashed blue lines) is shown in panels (d–h). The isosurfaces shown represent the HOMO → LUMO transition density on each molecule, scaled with the expansion coefficient of the time-propagated excitonic wave function eq 2 in the localized excitonic state basis, . At 255 fs, the exciton is localized on a single molecule (d). Following thermal excitation within the excitonic band the exciton temporarily expands over up to 10 molecules between 265 and 276 fs, i.e. becomes transiently delocalized (e–g), followed by de-excitation and relocalization on a single molecule at 285 fs (h), several lattice spacings apart from where the exciton started at 255 fs (as indicated by circles in dashed orange lines). Transient delocalization events make a large contribution to the diffusion constant, see Figure 5.

For analysis of the diffusion mechanism we consider three representative FE-SH trajectories (of a total of 900) and show the time-dependent IPR as a measure of the dynamical wave function delocalization in Figure 4a–c. We find that for each trajectory the average IPR is between 1.0 and 1.6 (lines in red), close to the average over the full swarm of trajectories, implying that the exciton is localized on a single molecule most of the time. As mentioned above, this is because reorganization energy is an order of magnitude larger than the largest excitonic couplings, and therefore localization is, on average, energetically preferred over delocalization (in analogy with small polaron formation for charge carriers).

However, frequent thermal excitations within the S_1_ excitonic band lead to transitions (“surface hops”) to higher-lying and delocalized excitonic states. These excitations and delocalizations of the excitonic wave function are usually short-lived, on the order of 10 fs and occur about every 100 fs. After de-excitation, the exciton relocalizes on one of the molecules it was delocalized over. If this molecule is not the same as the initial molecule it started from then the delocalization event contributes to exciton diffusion, see Figure 4d–h for an illustrative example. Importantly, we find that the extent of delocalization can vary substantially, from simple 2-state delocalization over neighboring molecules (see Figure 4b at 0–600 fs and Figure 4c) akin to small polaron or Marcus hopping, to more substantial delocalization events over 11 (Figure 4a) or even more than 20 molecules (Figure 4b, 800 fs).

**Figure 5:**
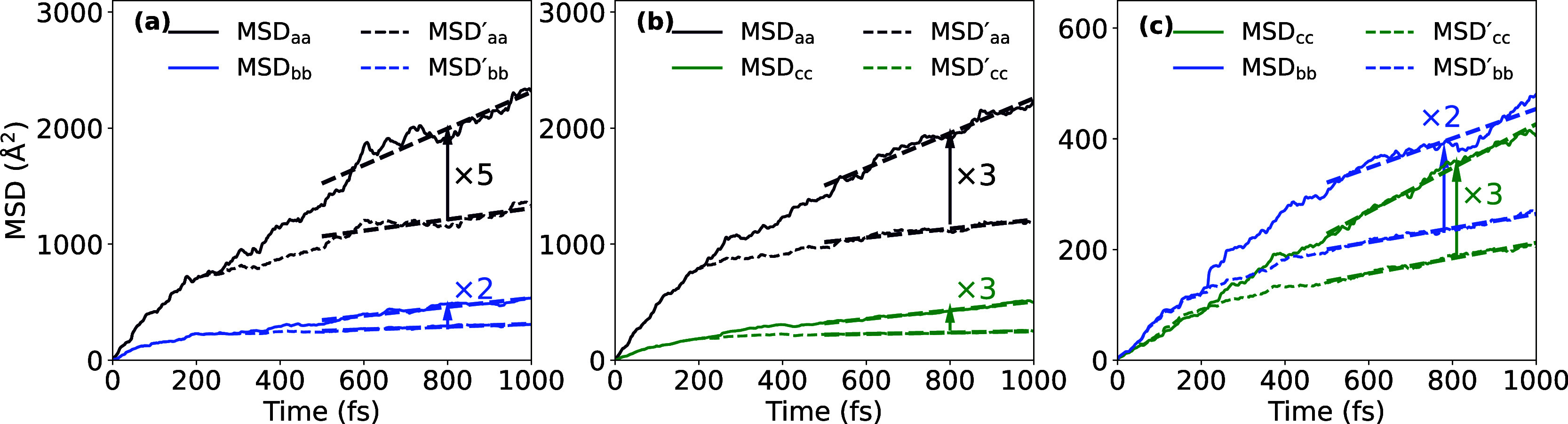
Impact of transient delocalization events on FE diffusion. The full MSD of the exciton wave function versus time, as obtained from FE-SH dynamics performed for the *a*–*b* (a), *a*–*c* (b), and *b*–*c* (c) crystallographic planes, are taken from Figure 3 (solid lines). The MSD with transient delocalization events excluded are shown in dashed lines, see text for details. The corresponding diffusion constants, , α = *a*, *b*, *c*, are obtained from linear fits of the MSDs between 0.5 and 1 ps and are summarized in Table 2. Note the sizable diffusion enhancement factors due to transient delocalization events.

In keeping with previous work,^10^ we define events where IPR(*t*) > ⟨IPR⟩ + 1 as “transient delocalization” to distinguish them from local or short-range hopping where, during the transition, IPR(*t*) = ⟨IPR⟩ + 1. To quantify the contributions of transient delocalization and local hopping events to the diffusion coefficient, we remove in the calculation of MSD all displacements of the exciton wave function that are a result of transient delocalization events. The resultant MSDs are then due to local hopping events only, see Figure 5 (dashed lines) and Table 2 for the corresponding diffusion constants excluding transient delocalization events, denoted , α = *a*, *b*, *c*. We find that the diffusion constants along the *a*-direction decrease by a factor of ∼4, and along the *b*- and *c*-directions by a factor of ∼3 when the transient delocalization events are excluded. The corresponding diffusion lengths decrease by approximately a factor of 2 in all three directions indicating that transient delocalization lead to a major increase in diffusivity.

## 4 Discussion

The computed diffusion constants for O-IDTBR are comparable with the ones computed previously for other single-crystalline nonfullerene acceptors.^10^ The largest component (*D*_*a*_ = 83 × 10^–3^ cm^2^ s^–1^) is half way between the values for perylene diimide (PDI, *D*_*a*_ = 26 × 10^–3^ cm^2^ s^–1^) and Y6 (*D*_*a*_ = 150 × 10^–3^ cm^2^ s^–1^), yet substantially larger than for typical organic crystals, for example, anthracene (*D*_*b*_ = 3.3 × 10^–3^ cm^2^ s^–1^). The larger diffusion constant compared to anthracene is mostly due to the smaller reorganization energy ( = 330 meV for O-IDTBR vs 561 meV for anthracene^10^) and the larger center of mass distances (*d*_com_ ≈ 1.5 nm in O-IDTBR vs 0.6 nm in anthracene) at comparable excitonic couplings along the high diffusion direction. By contrast, the smaller diffusion constant compared to Y6 (the NFA in OPV cells with the highest power conversion efficiency to date) is due to the smaller reorganization energy in Y6 ( = 250 meV) and the 2–3 times higher excitonic coupling values in Y6 at comparable center of mass distances. Nonetheless, the estimated exciton diffusion length for O-IDTBR (*L*_*a*_ = 97 nm) is longer than for Y6 (*L*_*a*_ = 87 nm)^10^ because of the longer exciton lifetime for O-IDTBR (561.5 ps^43^ vs 250 ps for Y6^60^). The results for the exciton diffusion lengths come with the caveat that the exciton lifetime was determined experimentally on thin film morphologies and may be different for single crystals.

In previous work, we carried out similar nonadiabatic dynamics simulations for electron transport in the same material,^45^ thus a comparison between exciton and electron transport is of interest. The most striking difference is that O-IDTBR is a 2D conductor for electrons (within the *a*–*b* plane) but a 3D “conductor” for excitons. Even within the 2D plane the anisotropy of electronic transport is significantly higher for electrons than for excitons, μ_*a*_/μ_*b*_ = 23 compared to *D*_*a*_/*D*_*b*_ = 5. The large anisotropy for electrons is mainly caused by the anisotropies in the electronic couplings: they are negligibly small along the *c*-direction and about a factor of 5 higher along *a* than along *b*. By contrast, the excitonic couplings in all three directions differ by no more than a factor of 2–3. The reason for this is that electronic couplings, being proportional to the overlap of frontier orbitals of neighboring molecules, decay exponentially with distance whereas excitonic couplings decay polynomially (∝*r*^–3^ in the transition dipole model). Thus, excitonic couplings are less sensitive to the different spacings between the molecules in different directions resulting in more isotropic transport. Another important difference is that electronic couplings are larger than the excitonic couplings along the high mobility/diffusivity direction and vice versa for reorganization energy, ⟨|*H*_*kl*_|^2^⟩^1/2^/ = 0.53 and ⟨|*V*_*kl*_|^2^⟩^1/2^/ = 0.055. The ratio for electrons exceeds the critical value of 1/2, above which small polarons are no longer stable; as a result the electron is delocalized over about 3 molecules on average (⟨IPR⟩ = 2.7). The ratio for excitons is well below this threshold resulting in localization on a single molecule on average, as mentioned above (⟨IPR⟩ = 1.2). Since diffusivity typically scales with delocalization, the diffusion constant for electron transport [*D*_*a*_(electron) = *k*_B_*T*μ_*a*_/*e* = 175 × 10^–3^ cm^2^ s^–1^] is about a factor of 2 larger than for excitons [*D*_*a*_(exciton) = 83 × 10^–3^ cm^2^ s^–1^].

Unfortunately, experimental estimates for diffusion coefficients or lengths in single crystals of O-IDTBR have not been reported, to our best knowledge. For neat O-IDTBR films an experimental diffusion length of ca. 10 nm has been reported,^35,61^ an order of magnitude shorter than our estimate for single crystals. Since , the diffusion constant in the thin film is then 2 orders of magnitude lower than in the crystal assuming similar exciton lifetimes. The strong decrease in diffusion in the thin film samples can be explained by the static disorder in thin-film morphologies which gives rise to large site energy differences and disruptions in the continuous excitonic coupling paths.^62−65^ Indeed, we found that electron mobility in thin film models of O-IDTBR is a total of 5–6 orders of magnitude lower than in O-IDTBR single crystals as a consequence of large static electrostatic site energy disorder (causing a 4–5 orders of magnitude reduction) and static electronic coupling disorder (causing a 1 order of magnitude reduction).^45^ Since excitons are bound electron–hole pairs they are less susceptible to electrostatic disorder than electrons, which could explain qualitatively why the decrease in diffusion constant for excitons is smaller (yet still very sizable) than for electrons.

## 5 Conclusions

In this work we have performed nonadiabatic dynamics simulation of exciton transport in the nonfullerene acceptor material O-IDTBR applying our recently introduced Frenkel exciton surface hopping (FE-SH) method. We found that the exciton in the crystal is, on average, localized on a single molecule due to the relatively small excitonic couplings with neighboring molecules and sizable reorganization energy. Though our simulations indicate that the transport mechanism is not what one would naively expect: in addition to local hopping we observe frequent transient delocalization events, in which the localized exciton expands over ten or more molecules for a short duration of time due to thermal excitations to higher-lying excitonic band states. These events significantly enhance the diffusion coefficient one would obtain for local hopping only, by a factor of 3–4. Similar results have been obtained before using nonadiabatic molecular dynamics for exciton transport in Y6 and the organic donor DCVSN5,^10^ as well as for charge transport.^19^ Notably, a quantum coherent transient localization mechanism has recently been reported for regioregular P3HT using full quantum simulations^6^ adding evidence that transient delocalization is a common transport mechanism for excitons and charges in ordered organic systems. It is very likely that transient delocalization is no longer active in application-relevant disordered thin films because the structural defects and electrostatic disorder will lead to increasing localization of higher-lying excitonic states so that slow hopping (with hopping rates slower than in the crystal) remains the only possible transport mechanism. This could be the reason why measured exciton diffusion lengths (10 nm) are an order of magnitude smaller than predicted here for the crystalline phase (100 nm). The present characterization shows that FE-SH is a very powerful and cost-efficient simulation method to obtain molecular-scale insight into the transport mechanism of excitons in molecular materials.

## Supporting Information Available

Isosurfaces of HOMO and LUMO orbitals of O-IDTBR, singlet excitation energies and reorganization energies calculated for different DFT functionals (PDF)
