# Supplementary material for: Exciton Transport in the Nonfullerene Acceptor O-IDTBR from Nonadiabatic Molecular Dynamics
Source: J Chem Theory Comput. 2024 Jul 5;20(14):6241–52. doi: 10.1021/acs.jctc.4c00605 (PMC11270823; doi:10.1021/acs.jctc.4c00605)
Supplement: Supplementary file 1 — ct4c00605_si_001.pdf [file ct4c00605_si_001.pdf]

Supporting Information

Exciton transport in the non-fullerene acceptor O-IDTBR from non-adiabatic molecular dynamics

Ljiljana Stojanovic,[†] Samuele Giannini,[‡] and Jochen Blumberger[*,†]

Department of Physics and Astronomy and Thomas Young Centre, University College London, London WC1E 6BT, UK., and Institute of Chemistry of OrganoMetallic Compounds, National Research Council (ICCOM-CNR), I-56124 Pisa, Italy.

E-mail: j.blumberger@ucl.ac.uk

[*]To whom correspondence should be addressed

[†]Department of Physics and Astronomy and Thomas Young Centre, University College London, London WC1E 6BT, UK.

[‡]Institute of Chemistry of OrganoMetallic Compounds, National Research Council (ICCOM-CNR), I-56124 Pisa, Italy.

We tested the performance of three density functionals for the TDDFT calculation of the 3 lowest-lying excited states of the O-IDTBR molecule, CAM-B3LYP, ωB97X-D and M06-2X using the 6-31G(d,p) basis. The S_1-S_3 states are computed at O-IDTBR geometry extracted from the crystal structure.[1] We have also computed the reorganization energies for the exciton transport applying the four-point method on an isolated molecule (as explained in the manuscript). The results are summarized in Table 1. The energies of the S_1-S_3 states and their oscillator strengths obtained with three tested functionals are very close in values (the maximum deviation in energies corresponding to the same state is < 0.2 eV). The first two excited states are separated by $\sim$0.35 eV, while the third excited state lies $\sim$0.5 eV above the S_2 state. The reorganization energies for the exciton transport computed at the CAM-B3LYP/6-31G(d,p) and M06-2X/6-31G(d,p) are in a relatively good agreement (334 and 375 meV, respectively), differing by only $\sim$40 meV. The ωB97X-D functional predicts a somewhat larger reorganization energy of 424 meV, yet the ωB97X-D functional usually requires fine tuning of the ω-parameter which we have not done here. We opted to use CAM-B3LYP mainly to stay as consistent as possible with a previous parametrization of the force field for the ground state of O-IDTBR.[2]

Table 1: The energies (E in eV) and oscillator strengths of the first three excited states of the O-IDTBR molecule. The reorganization energies for exciton transport for the S_1 excited state (λ in meV) are also provided.

Functional	$E(S_1)$	$f(S_1)$	$E(S_2)$	$f(S_2)$	$E(S_3)$	$f(S_3)$	λ
CAM-B3LYP	2.28	2.71	2.61	0.00	3.18	0.00	334
ωB97X-D	2.37	2.83	2.71	0.00	3.22	0.00	424
M06-2X	2.26	2.71	2.60	0.00	3.05	0.00	375

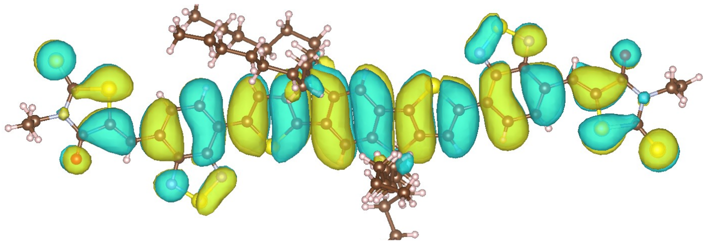

HOMO

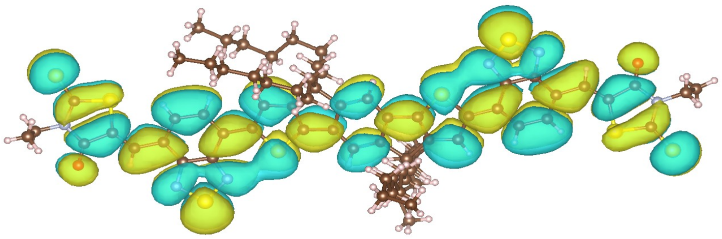

LUMO

Figure 1: HOMO and LUMO of the O-IDTBR molecule computed at the CAM-B3LYP/6-31G(d,p) level of theory at the geometry extracted from the crystal structure.[1]

References

(1) Bristow, H.; Thorley, K. J.; White, A. J. P.; Wadsworth, A.; Babics, M.; Hamid, Z.; Zhang, W.; Paterson, A. F.; Kosco, J.; Panidi, J.; Anthopoulos, T. D.; McCulloch, I. *Adv. Electron. Mater.* **2019**, *5*, 1900344.

(2) Gertsen, A. S.; Sorensen, M. K.; Andreasen, J. W. *Phys. Rev. Materials* **2020**, *4*, 075405.
